# Development of the social burnout scale for college students

**DOI:** 10.3389/fpsyg.2024.1295755

**Published:** 2024-03-21

**Authors:** Jing Wang, Chang Liu, Longling Chen, Qiuyue Liao, Guoqin Liu

**Affiliations:** ^1^School of Management, Zunyi Medical University, Zunyi, China; ^2^School of Business, Macao University of Science and Technology, Macao, China

**Keywords:** social burnout, grounded theory, scale development, social skill, interpersonal trust

## Abstract

Although fruitful achievements have been explored about job burnout, little is known about burnout in the field of social interaction among college students. To address this limitation, this study defined the concept of Social Burnout and developed a measurement tool for it. The study adopted the method of combining qualitative research with quantitative research. After the qualitative study, we gathered examples of social burnout and finished item writing. Using convenient sampling and theoretical sampling methods, six different samples were recruited for reliability and validity testing. Confirmatory factor analysis (CFA) revealed the scale’s two-factor structure: emotional exhaustion and depersonalization. Cronbach’s alpha measured the internal consistency of the social burnout scale (SBS), which was excellent (Cronbach’s alpha of emotional exhaustion = 0.94; depersonalization = 0.82; the overall = 0.92). Susequently, the method of calculating AVE and CR evaluated the scale’s convergent and discriminant validity, which were relatively good (AVE of emotional exhaustion = 0.60, depersonalization = 0.59; CR of emotional exhaustion = 0.93, depersonalization = 0.81). Then, regression analysis verified the nomological network and criterion-related validity (*r* = −0.30, *p* < 0.01; r = −0.39, *p* < 0.01; *β* = −0.25, *p* < 0.01). The SBS was shown to be a reliable and appropriate measure for assessing students’ social burnout. Furthermore, the SBS is recommended for use in academic research and by healthcare professionals to measure students’ social distress. Further validation studies of this scale are needed in other cultural contexts.

## Introduction

The term “burnout” described a social problem that had existed for a long time. Initially, burnout specifically referred to job burnout. Namely, in the professional field of serving people, a group of negative symptoms caused by the insufficient work resources of individuals to meet their work requirements, such as excessive consumption of emotional resources, loss of enthusiasm for work, and decreased sense of accomplishment at work, etc. Nowadays, burnout has become a striking social and health problem, and this field of research is gradually expanding around the world. Research has begun in other professional fields, especially recently, with an increase in studies involving college graduates and medical students (Rosales-Ricardo et al., 2021; Schaufeli et al., 2022). Subsequently, scholars argued that burnout might occur in all areas that bring people a sense of value and meaningfulness, and put forward the concept of marriage burnout (Pines, 1987), parental burnout (Wang et al., 2021), social media burnout (Lee et al., 2016; Technopedia, 2017), study-related burnout (Schaufeli and Taris, 2005) and so on.

The current literature of burnout presents three components as emotional exhaustion, depersonalization and low personal realization (Maslach and Jackson, 1981a,b). And it can be seen that, research on burnout points to a common language (in terms of measurement) that comes from the Maslach Burnout Scale (MBI), which has been, and is, the primary measure of burnout (Rosales-Ricardo et al., 2021). It was said that about 90% of empirical papers have used this measurement (Schaufeli et al., 2009). However, several researchers have argued that the core dimensions of burnout are exhaustion and depersonalization (Densten, 2001; Schaufeli et al., 2009; Beer and Bianchi, 2017).

Despite a large amount of evidence shows that college students may suffer from burnout, an important kind of burnout has been neglected, that is burnout occurs in interpersonal communication. It is especially easy to happen in college students. Plenty of evidence shows that more and more students are facing serious interpersonal communication problems. If prolonged, these students maybe in a risk of stress or burnout (Schaufeli et al., 2009). For example, as is shown by the “2010–2011 College Student Mental Health Survey Report,” the main causes of college students’ psychological problems are interpersonal pressure and weak ability to deal with emotional problems. Besides, a survey of 2,006 respondents conducted by the China Youth Daily Social Survey Center in 2020 showed that 63.90% of the respondents’ WeChat moments were set to be visible for 3 days because they believed that using this function could avoid unnecessary troubles or protect themselves. However, no concept that can summarize this phenomenon has been explored in existing research.

The standardization of the Maslach burnout Inventory-Student Survey (MBI-SS) by Schaufeli et al. (2022) makes it possible to operationalize various burnout. This makes it possible to measure burnout outside the professional field by defining its dimensions with reference to student research. Its application suggests that a significant proportion of students reflect exhaustion, as well as attitudes of disinterest, and feeling of incompetence as students. The international scientific literature discusses the prevalence of this syndrome in the college student population in different ways, largely based on the diversity of tools used for assessment. Due to the heterogeneity of these studies, this leads to a certain complexity in comparing and analyzing the results of these studies. Unfortunately, most of these tools have not been developed and validated for the student population. For example, Xue (2011) proposed the concept of interpersonal relationship burnout of college students and defined it as a symptom of emotional exhaustion, depersonalization and reduced personal accomplishment in the process of interacting with others. This concept is similar to the core concept of this study, social burnout. However, the study of interpersonal burnout has been limited to some extent due to the lack of a specific scale.

This article intentions to provide a synopsis of the phenomenon stated above, its definition, construct and measurement. Based on literature review and interview data, this study will propose the concept of social burnout, develop a measurement tool for it, and further verify the reliability and validity of the social burnout scale, in order to lay a foundation for future research.

## Literature review

### Concept and measurement of burnout

It was Freudenberger (1974), an American clinical psychologist, first explored the concept of job burnout. Freudnberger first defined burnout as “exhaustion due to excessive demands on energy, vitality, or resources” (Freudenberger, 1974) and applied the concept to professions such as teachers and nurses to describe the emotional exhaustion experienced by these groups as a result of working too long and hard hours. However, in numerous literatures, the most widely cited was Maslach and Jackson’s definition, that was “A symptom of emotional exhaustion, depersonalization, and reduced personal achievement of individuals in human-oriented professional fields” (Maslach and Jackson, 1981a,b).

With the gradual deepening of the research, Maslach gave an operational definition of job burnout which consist of three dimensions, namely emotional exhaustion, depersonalization, and low personal accomplishment. He argued that emotional exhaustion was a core symptom of job burnout. It was an exhaustion of emotional resources (i.e., an unwillingness to give more affection), which could lead to a state of apathy. Depersonalization was the development of a cold, negative attitude toward others and an unwillingness to face people or things related to work. Low personal accomplishment referred to the tendency to evaluate oneself negatively and workers holding the opinion that his/her work was meaningless.

As research progresses, the scope of the concept of job burnout has been modified and expanded to a wider range of occupations. The revised concept also included three dimensions of exhaustion, cynicism and decreased professional self-efficacy. Among them, exhaustion was defined as the loss of psychological resources, and cynicism was defined as not caring about one’s work and have a sense of distance, that was, job slack.

Based on the definition of the three-dimensional model, Maslach and Jackson (1981a,b) developed the Maslach Burnout Inventory (MBI). Since the early 1980s, the measurement of burnout has been dominated by it. It was said that about 90% of empirical papers have used this measurement (Schaufeli et al., 2009). There are three versions of this scale, the MBI-Human Service Survey (MBI-SS), the MBI-Educators Survey (MBI-ES), and the MBI-General Survey (MBI-GS).

MBI-GS is more universal and can be applied to a larger population. It is consisted of 16 items, measured from three defined dimensions (exhaustion, cynicism and decreased professional self-efficacy). MBI-GS adopts the 7-point Likert scoring method, requiring subjects to rate the given items according to their true feelings on a 7-point scale ranging from 0 to 6. A typical example of the exhaustion dimension is: “I feel burned out from my work.” Example-item of cynicism is: “I have become more cynical about whether my work contributes anything.” Finally, example-item of professional self-efficacy is: “In my opinion, I am good at my job.”

As for the measurement of student samples, the most commonly used tool internationally is Schaufeli et al. (2022) MBI-SS (e.g., Ilic et al., 2017; Portoghese et al., 2018; Liu et al., 2023), which has three dimensions: emotional exhaustion, cynicism, and academic self-efficacy.

In contrast, the MBI (Maslach and Jackson, 1981a,b), which has three dimensions: exhaustion, depersonalization, and reduced personal accomplishment, has also been frequently used for the diagnosis of student populations (Obregon et al., 2020; Pitanupong et al., 2023).

In addition, there are other measuring tools such as the Copenhagen Burnout Inventory (Kristensen et al., 2005), the Oldenburg Burnout Inventory (Halbesleben and Demerouti, 2005; Loscalzo et al., 2023; Macaron et al., 2023), and so on.

### Social burnout

Considering that burnout may occur in a variety of fields that can bring people a sense of value and meaning, and there is a phenomenon of social avoidance and disinterest in social interaction among college students. So, the research question is whether there is social burnout among college students? How to define and measure social burnout? Unfortunately, there are few researches on social burnout in academic circles, which neither provide direct reference for this study nor measure the social burnout of college students in real life. But we can take a cue from burnout and related concepts. Job burnout is the physiological, psychological and behavioral burnout that occurs in the process of work. While the related concept such as social media burnout refers to the psychological and behavioral burnout of users during human-computer interaction. Social burnout arises from the interaction between people. Although it is different from job burnout and social media burnout, it also has some similarities with them. For example, they all include emotional and cognitive components.

As was explained by Maslach et al. (2001), interpersonal stressors were also possible cause of burnout. Therefore, this study proposed the construct of social burnout based on the related literature of burnout. We set out to systematically develop and validate a set of social burnout scales following the advice of Hinkin (1995, 1998). Using grounded theory, we conducted in-depth interviews with college students, dug into the forms of social burnout, refined the conceptual structure of it, and completed connotation definition and scale development of social burnout. The whole process was separated into two studies, one is a qualitative study, and the other is the scale development study. Refer to Table 1 for an overview of study phases and samples.

**Table 1:** Overview of study phase and samples.

Phase description	Samples used
*Study 1-Qualitative study of social burnout* Phase 1 preliminary interview Purpose: to form a formal interview outline Phase 2 formal interview Purpose: gather examples of social burnout to guide item writing	1 2
*Study 2-Scale development and psychometric characteristics* Phase 1: item generation and reduction Purpose: generate an item pool and then simplify the items by means of content validity assessment, exploratory factor analysis, and confirmatory factor analysis	3 4 5
Phase 2: internal consistency Purpose: assess the internal consistency	4 + 5
Phase 3-convergent and discriminant validity Purpose: demonstrate significant correlations between the social burnout scales and related constructs, and weak or non-significant correlations between the social burnout scales and theoretically unrelated constructs. Verify distinctness between the social burnout scales and other scales.	4 + 5
Phase 4-nomological network and criterion-related validity Purpose: test for significant relationships between the social burnout scales and other constructs based on theoretical predictions.	6

Sample description		*N*	Date source	Location
Sample 1		23	3 Ph.D. students and 20 college students	China
Sample 2		65	Students from a university in southwest China	China
Sample 3		10	Ph.D. students majoring in organizational behavior and human resource management	China
Group 1	Sample 4	211	A medical college in southwest China	China
	Sample 5	212	A medical college in southwest China	China
Group 2	Sample 6	213	A medical college in southwest China	China

## Study 1: qualitative study of social burnout

The objectives of this study were to gather examples of social burnout which would guide concept definition and item development. This study adopted the method of combining qualitative research with quantitative research. The reason lies in that, the mixed use of multiple methods can make the research conclusions more convincing. According to Yin (2014), “those case studies using multiple sources of evidence were rated more highly, in terms of their overall quality, than those that relied on only single sources of information” (page. 131). Following Corbin and Strauss (2008), the steps of qualitative research include data collection, open coding, axial coding, selective coding, and theoretical saturation test. Data coding was guided by the grounded theory, and quantitative research mainly followed the scale development procedure of Hinkin (1995, 1998).

After the research question was established, we started our research by conducting a qualitative pilot study. The aim of this research was to gather examples of social burnout which would guide concept definition and items development. In this study, face-to-face semi-structured interviews were adopted to obtain relevant data, and the coding program of Strauss (1987) proceduralised grounded theory was adopted to conduct coding analysis of the data. The interviewers were two authors (the first author and the corresponding author) of this article. Their research area involved in organizational behaviors and they were familiar with the topic of burnout. They have experienced social burnout or have observed it in their daily lives. They have conducted interviews many times and is proficient in interviewing techniques. The specific steps were open coding, axial coding, and selective coding. The encoding rules are unified as “date + name + sentence,” such as 20220102ZY001.

### Materials and methods

#### Data collection

There can be multiple sources of information for the analysis of grounded theory, from primary information such as interviews with selected research samples, as well as secondary information such as relevant reports, online sources, literature, and publications. In order to ensure a variety of data sources and form triangulation between data, students of different grades were selected for the interview, and secondary data from literature and third-party reports (e.g., China Youth Daily Hwakth Survey Report) were collected as supplements. The data used in this study was collected from November 2022 to December 2022 by the corresponding authors and the fourth author of this study. They are well-trained and experienced researcher, two of whom have doctorates in management. They have previously published an article on scale development. As suggested by Yin (2014), in order to ensure the methodological rigor of the case study process, all team members received uniformly training before data collection.

Related literature was searched through Web of Science and China National Knowledge Infrastructure (CNKI) by combining key words such as “burnout,” “social burnout,” “student burnout,” “interpersonal burnout,” etc. Finally, 2 articles retrieved under the index of CNKI and 25 articles retrieved under the index of SSCI were selected as qualitative information.

After the literature collection was completed, we began the formal semi-structured interviews.

Firstly, before the formal interview, we needed to make an interview outline. Sample 1 (3 Ph.D. students majoring in psychology and 20 college students) helped us achieve this goal. After the research topic and research objectives are determined, we invited three Ph.D. students majoring in psychology to work out a preliminary interview outline, and each student could get 10 yuan (RMB) as payment. As a result, three questions were identified, namely: “(a). What is your understanding of social burnout? (b). Have you ever experienced social burnout? (c). What are the possible manifestations of social burnout?” Then, in order to check whether the interview outline is appropriate and whether it needs to be supplemented and modified, we conducted a preliminary interview. We invited 20 college students to participate in the preliminary interview, explained our research intention to them, asked them to give suggestions and opinions on the interview outline, and optimized the interview outline according to their feedback to form a formal interview outline. The participants were convenience samples, and they completed the study in exchange for payment (5yuan (RMB) per person). Face-to-face interviews were conducted with the 20 participants. Each person’s interview time was limited to about 30 min. During the interview, respondents were encouraged to express their opinions and thoughts and actively share examples. We take the interviewees’ own learning and life experiences as the starting point to reduce their resistance, and gradually lead to the related issues of social burnout. During the interview, we found that interviewees often mentioned the antecedents and consequences of social burnout. In order to obtain more abundant data, we added the antecedents and consequences of social burnout into the interview outline. Therefore, the formal semi-structured interview questions were “(a). What is your understanding of social burnout? (b). Have you ever experienced social burnout? (c). What are the possible manifestations of social burnout? (d). What are the possible antecedents of social burnout? (e). What are the possible consequences of social burnout?”

Next, the formal semi-structured interviews were conducted in this study. Our initial goal was to interview 16–24 participants as Hennink et al. (2017) suggested that 16 to 24 interviews were needed to reach meaning saturation. However, theoretical saturation was not reached until the 65th person was interviewed. That was, no additional issues or insights emerged and all relevant conceptual categories had been identified at that point. So, sample 2 was a sample of 65 students from a university in southwest China participated in the survey, and we got this sample using theoretical sampling. The 65 participants completed the study in exchange for payment (5 yuan (RMB) per person). The reason for choosing these samples is that, these students’ WeChat moments were set to be visible for only 3 days or they have a low frequency of updates in WeChat moments. The total sample included 30 males and 35 females. Their average age was 22.02 ± 1.05. There were 12 sophomores (18.5%), 47 juniors (72.3%), and 6 seniors (9.2%). Participants were asked to remember and describe whether they had experienced social burnout, how did they understand this concept, the forms, causes and consequences of social burnout. Each person’s interview time ranged from 10 min to 25 min. All interviews were conducted in Mandarin, and approximately 12,000 words of interview data were compiled as a result.

Finally, the researchers archive all the data collected, both primary and secondary, to form a database needed for subsequent coding analysis. In the coding process, interview data are mainly used, supplemented by existing research articles collected.

In order to ensure data saturation, 2/3 of the data was used for coding analysis, and the remaining data was used for comparison and saturation test.

#### Data analysis process

Grounded theory is a type of qualitative research, and its main purpose is to establish theories based on empirical data (Glaser and Strauss, 1968). It is a bottom-up approach to build a theory, starting directly with practical observations, generalizing empirical generalizations from the primary data, and then rising to a systematic theory. Grounded theory is applicable to exploratory research in relatively new fields. Therefore, this study adopted grounded theory for procedural coding analysis, deeply depicted qualitative information, and revealed the connotation and conceptual structure of social burnout. According to the grounded theory, this study followed a systematic process of posing questions, collecting data, analyzing data, and building theories, making the research contain abundant information and more closely related to management practices.

#### Open coding

Open Coding is an operational process of “crushing” the collected material, giving it a conceptual label, and then re-combining it with a new formula to define the concept and discover the category. First, the researchers randomly selected three primary data for pre-analysis and reached a consensus on the coding standard. Subsequently, the primary data were coded back-to-back by two researchers (the co-author and the third author) to conceptualize and categorize the primary data. The detailed steps are as follows: (a) Label the data, extract the corresponding concept, and define the phenomenon; (b) Carry out in-depth analysis of the concept to dig out more general categories; (c) Give the categories accurate names; (d) Explore and summarize the nature and dimension of category. After these steps were completed, we finally got 14 initial concepts according to the principle of similarity of meaning. Due to the limited space, only partial examples of open coding are listed in this study, as shown in Table 2.

**Table 2:** The results of open coding.

Typical statement	Initial concept	Initial category
I do not like to communicate with people. I feel uncomfortable communicating with people	Dislike communicating with others	Dislike and uncomfortable
I feel tired and run away from people	Exhaustion and escape	Emotional exhaustion and behavioral avoidance
I began to perfunctory friends, did not care about friends’ feelings, and even wanted to stay away from my friends	perfunctory and neglect friends, stay away from friends	Be perfunctory, indifferent and stay away
Feeling tired, showing a strong aversion to socializing, wanting to do your own thing and not wanting others to know what you are doing	Tiredness, disgust and invisibility	Emotional exhaustion and behavioral avoidance
Feel that communication with people is not interesting, unnecessary, do not want to answer others, addicted to one’s own thoughts	Boring, unnecessary, and stubborn	Attitudinal aversion

#### Axial coding

The main task of axial coding is to establish connections between various independent categories, reveal the potential logical relations between categories, and develop the main categories and sub-categories. The detailed content and connotation of the categories are shown in Table 3.

**Table 3:** The results of axial coding.

Main category	Sub-category	Initial category	Category connotation
Emotional exhaustion	Uncomfortable and exhaustion	Dislike and uncomfortable	Being emotional overextended and exhausted during social interaction
	Lack of enthusiasm	Emotional exhaustion and behavioral avoidance	
Depersonalization	Indifference	Be perfunctory, indifferent and stay away	An unfeeling and impersonal response towards others during social interaction
	Distrust	Distrust of others	

#### Selective coding

Selective coding is to integrate and refine the content formed by the axial coding once again, that is, to dig out the “core category” that can lead other categories from the main category, and use the typical model to systematically link it with other categories, analyze and verify its linkage. As a result, a theoretical framework is formed by developing a “Story Line” to depict all contextual conditions and behavioral phenomena. According to the research purpose, the original qualitative data and open coding results were re-examined, and the relationship between the core categories was established. After the step by step procedure of the study, it was found that the core concept of social burnout consisted of two main categories: emotional exhaustion and depersonalization.

#### Theoretical saturation test

When the semantic analysis and conceptual reconstruction of new qualitative data do not produce new categories and no new relationships between categories, it can be considered that the results of grounded theory have reached theoretical saturation. In this study, another 1/3 of the interview data was used for theoretical saturation test. After in-depth analysis and comparison, no new concepts and categories that could affect the core category were discovered, and we had fully understood the issues (Hennink et al., 2017). Therefore, it can be considered that the model reached theoretical saturation.

### Findings

After the interview, we got 334 detailed responses (103 understandings of the concept, 126 forms of social burnout and 105 causes of social burnout) in total. Each response was evaluated by two independent raters (two of the authors of this article) to determine if it met the definition of social burnout. Responses that did not clearly describe the forms of burnout during social interaction were considered not to be social burnout and were removed, e.g., “information overload” was removed. All rater disagreements were completely resolved through rater discussion.

A total of 139 cases were collected, with an average of 2 case per person, including 50 understandings of the concept, 55 forms of social burnout and 34 causes of social burnout.

Combining the qualitative information obtained from interviews with relevant literature, a two-dimensional structure of social burnout was constructed according to the structured grounded theory analysis process, which were emotional exhaustion and depersonalization.

Our findings suggested that there was indeed a phenomenon of social burnout among college students, which manifested itself in a variety of forms, such as disinterest in the people around oneself, feeling tired of interacting with others, trying to avoid crowds, and so on. Based on the above findings, and referencing Maslach et al. (2001) definition of burnout, we define social burnout as a symptom of emotional exhaustion and depersonalization in the process of interacting with others. It is the tendency of social groups to become psychologically tired and to avoid and slack off in behavior due to individual, social and other factors. Emotional exhaustion refers to the physical and emotional overloads that result from interactions with others. Depersonalization refers to a skeptical and cold attitude towards others, and the loss of the personal element in dealing with individuals.

## Study 2: scale development and psychometric characteristics

### Phase 1: item generation and reduction

Firstly, by analyzing the original data and the contents of the theoretical construction part, the relevant material statements are extracted from the dimensions of the conceptual framework. Secondly, this study also draws on existing literature and scales, such as job burnout scale and social media burnout scale. According to the concept of social burnout, the researchers combined the original data to write 38 semantically clear declarative sentences as the initial item pool. Of these, three items (E1, E2 and E3) were derived from MBI-General Survey (MBI-GS; Schaufeli et al., 1996), and two items (E12 and E13) were derived from the social media burnout scale (Lee et al., 2016; Xu and Wang, 2023). The remaining items were all derived from grounded research. After repeated discussion by the authors of this article, the items with unclear semantics or repetitive expressions were deleted or merged until there was a consensus. After a preliminary cleaning, 30 items were retained (See Table 4).

**Table 4:** Original measurement items of the social burnout scale.

Dimension	Number	Item
Emotional exhaustion	E1	Interpersonal communication is a hassle
	E2	Interpersonal interactions make me tired
	E3	Interpersonal interaction makes me feel exhausted
	E4	I have resistance to interpersonal interactions
	E5	I hate interpersonal interactions.
	E6	I just want to immerse myself in my own world.
	E7	I feel comfortable shutting myself off
	E8	I do not want to communicate with others
	E9	Pandering to others wears me out
	E10	I get no psychological satisfaction in interpersonal communication
	E11	I belong to the role of being left out in human interactions
	E12	Interacting with people exposes privacy
	E13	Interacting with people is a waste of time
	E14	Interacting with people disappoints me
	E15	I do not get happy when I interact with people
	E16	There’s no need to meet too many people
	E17	My social network is big enough now that there is no need to expand it
Depersonalization	D1	I do not want to pander to others
	D2	I often put my friends off
	D3	I often do not care about my friends’ feelings
	D4	I often intentionally do not respond to my friends’ messages
	D5	I am cold to others
	D6	The people around are not trustworthy
	D7	I often do not fit into the collective
	D8	I always run away from crowds
	D9	When interacting with people, I do not always express my opinion
	D10	I do not want to share my daily life with others
	D11	I do not want to deal with people
	D12	I resist socializing internally
	D13	I want to stay away from or give up certain social interactions

### Content validity

As suggested by Hinkin (1998), this study assessed the content validity of the social burnout scale using a substantive validity item-sort task (Anderson and Gerbing, 1991). The purpose was to identify and retain items with substantive validity and drop items without it. Participants were given the whole items of an intended construct and several other constructs. For each item, participants were asked to choose which from a list of constructs the item best represents. After the participants assign each item to a construct, a significance test is required to determine whether an item was assigned to the intended construct more so than an acceptable level of chance. Anderson and Gerbing (1991) suggested a specific formula to determine statistical significance.

First, they proposed the substantive-validity coefficient (C_sv_), which indicated “the extent to which respondents assign an item to its posited construct more than to any other construct” (Anderson and Gerbing, 1991). C_sv_ was defined as follows:


$$C_{sv}=\frac{n_{c}-n_{o}}{N}$$

Where n_c_ represented the number of participants assigning a measure to its intended construct, n_o_ represents the highest number of assignments of the items to any other constructs.

Second, they suggested to calculate the critical value of c_sv_, denoted as $\bar{\;c}$_sv_, was defined as follows:


$${\bar{c}}_{sv}=\frac{n_{c}-\left(N-n_{c}\right)}{N}$$

where n_c_ and N were defined as before.

According to Anderson and Gerbing (1991), “if an item’s C_sv_ values is equal or greater than the $\bar{\;c}$_sv_, then it should be retained for further analysis.”

#### Participants and procedure

For sample 3, participants were 10 Ph.D. students majoring in organizational behavior and human resource management (50% female; mean age = 26.80 years). They completed the study in exchange for payment (5 yuan (RMB) per person). We chose these participants for the following reasons: first, it was suggested that “in the content validity, it may be appropriate to use a small sample of students as this is a cognitive task not requiring an understanding of the phenomena under examination” (Anderson and Gerbing, 1991; Hinkin, 1998); next, these participants were familiar with the subject and were able to make professional judgments. Participants were given the list of 30 items and five construct definitions. Participants were then asked to choose the most appropriate construct for each item. The five constructs were social fear, social anxiety, social media fatigue and job burnout.

#### Results

As suggested by Anderson and Gerbing (1991), we calculated and compared the C_sv_ value and $\bar{\;c}$_sv_ value. Finally, 10 items were removed and 20 items were retained. Finally, we got a 20-item social burnout measurement scale, as shown in Table 5.

**Table 5:** The refined social burnout scale.

Dimension	Number	Item
Emotional exhaustion	E1	Interpersonal communication is a hassle
	E2	Interpersonal interactions make me tired
	E3	Interpersonal interaction makes me feel exhausted
	E4	I have resistance to interpersonal interactions
	E5	I hate interpersonal interactions
	E6	I just want to immerse myself in my own world
	E7	I feel comfortable shutting myself off
	E8	I do not want to communicate with others
	E9	Pandering to others wears me out
Depersonalization	D1	I do not want to pander to others
	D2	I often put my friends off
	D3	I often do not care about my friends’ feelings
	D4	I often intentionally do not respond to my friends’ messages
	D5	I am cold to others
	D7	I often do not fit into the collective
	D8	I always run away from crowds
	D10	I do not want to share my daily life with others
	D11	I do not want to deal with people
	D12	I resist socializing internally
	D13	I want to stay away from or give up certain social interactions

### Exploratory factor analysis

To explore the factorial structure and item loading of the scale, an exploratory factor analytical (EFA) approach was conducted using sample 4. As Watkins (2018) suggested, it was necessary to verify that the measured variables were sufficiently intercorrelated to justify factor analysis. A possible subjective method was to examine the correlation matrix. “A sizable number of correlations should exceed ±0.30 or EFA may be inappropriate.” At the same time, there were objective method by calculating Bartlett’s test of sphericity, which statistically tests the hypothesis that the correlation matrix contains ones on the diagonal and Zeros on the off-diagonals. To justify the application of EFA, the chi-square value must be statistically significant. Besides, large sample size may make the Bartlett test sensitive to deviations from randomness, so it’s necessary to supplement a measure named Kaiser-Meyer-Olkin (KMO; Kaiser, 1974). The KMO value ranged from 0.00 to 1.00, and the closer the value is to 1, the more common factors between the variables, the more suitable for EFA. In this study, The Bartlett’s sphericity test and the KMO value were calculated to check whether the sample data was suitable for factor analysis. As suggested by Kaiser (1974), if the KMO value was larger than 0.60 and the Chi-square of Bartlett sphericity evaluation was significant, it indicate that meaningful factorial structures could be extracted from the data. Thereafter, principal component analysis and maximum variance rotation method were used to extract factors with eigenvalues greater than 1.

#### Participants and procedure

This study employed both cross-sectional data and longitudinal data gathered between January 2023 to June 2023. Participants were 644 students from a medical college in southwest China who completed the study in exchange for practical credits. All participants who participate in the survey will receive 2 practical credits. However, eight were dropped due to incorrectly answering a simple attention check item included in the study, leaving a final sample of 636. This sample consisted of Group 1 and Group 2, comprising a total of 217 male and 419 female participants. There were 169 freshmen (the first year of college; 26.60%), 316 sophomores (the second year of college; 49.70%), 104 juniors (the third year of college; 16.40%) and 47 seniors (the fourth year of college; 7.40%). There were 558 (87.70%) students majoring in medicine and 78 (12.30%) students majoring in non-medicine. Following the approach of many scholars (Li et al., 2023; Lin et al., 2023; Tan, 2023), Group 1 (n = 423 after 5 were dropped) was randomly divided into two parts and named Sample 4 and Sample 5, each with a certain purpose (see Table 1). Characteristics of the full sample are shown in Table 6. Sample 4 consisted of 211 questionnaires (3 were dropped), which were used for item analysis and exploratory factor analysis (EFA). Sample 5 consisted of 212 questionnaires (2 were dropped), which were used for confirmatory factor analysis (CFA) and reliability analysis. Group 1 were used for convergent validity, discriminant validity. Group 2 consisted of 213 questionnaires (sample 6; 3 were dropped) were used for nomological network and criterion-related validity analysis.

**Table 6:** Characteristics of the sample.

	Total	Group 1		Group 2
		Sample 4	Sample 5	Sample 6
Number (n)	636	211	212	213
% female	77.20	77.20	79.70	74.60
% freshmen	26.60	25.60	25.90	28.20
% Medical profession	87.70	85.30	88.20	89.70

#### Results

Firstly, the Kaiser-Meyer-Olkin (KMO) value and Bartlett sphericity test were calculated to check whether the sample data was suitable for factor analysis. The value of KMO was 0.94, and the value of Bartlett sphericity test was 4781.64 (*p* < 0.001), meeting the requirements for EFA. Second, principal component analysis and maximum variance rotation method were used to extract factors with eigenvalues greater than 1. Seven items with cross loading and factor loading less than 0.50 were deleted, leaving 11 items. According to Watkins (2018), there were no hard-and-fast rules to identify those items that clearly load on a single appropriate factor, but the 0.40 criterion level were most commonly used in judging factor loadings as meaningful. However, in this study, we excluded the items with factor loading less than 0.50, because it’s said that, “an appropriate loading of greater than 0.40 and/or a loading twice as strong on the appropriate factor than on any other factor” (Hinkin, 1998). Besides, we also calculated the Pearson correlation coefficients of the remaining 11 items and found that they were all greater than 0.3. This result once again proved that EFA was reasonable. Results of EFA were presented in Table 7. The cumulative variance contribution rate of the two dimensions was 56.13%, and the factor load distribution of each item ranged from 0.70 to 0.88. According to the meaning of each item, the two dimensions were named as emotional exhaustion and depersonalization.

**Table 7:** The results of EFA.

Items	Dimensions	
	Emotional exhaustion	Depersonalization
E2 Interpersonal interactions make me tired	0.85	
E6 I just want to immerse myself in my own world	0.85	
E5 I hate interpersonal interactions	0.85	
E7 I feel comfortable shutting myself off	0.84	
E4 I have resistance to interpersonal interactions	0.83	
E3 Interpersonal interaction makes me feel exhausted	0.82	
E1 Interpersonal communication is a hassle	0.77	
E8 I do not want to communicate with others	0.71	
D3 I often do not care about my friends’ feelings		0.88
D2 I often put my friends off		0.84
D4 I often intentionally do not respond to my friends’ messages		0.84

### Confirmatory factor analysis

A confirmatory factor analysis (CFA) was conducted using AMOS 21.0 and sample 5 to examine the structural equation model of social burnout. The following well-known fit indices were used to evaluate the goodness of fit of the CFA: comparative fit index (CFI), Tucker Lewis Index (TLI), Normed Fit Index (NFI), Root Mean Square Error of Approximation (RMSEA), Standardized Root Mean Square Residual (SRMR), and the chi-square test. They were accepted if the values greater than 0.90 for the CFI and TLI indices (Hu and Bentler, 1999). However, they were considered to indicate a good fit to the data if values of RMSEA and SRMR smaller than 0.08 and 0.06, respectively (Hu and Bentler, 1999).

#### Participants and procedure

As discussed above, we used Sample 5 for confirmatory factor analysis (CFA).

#### Results

**Figure 1:**
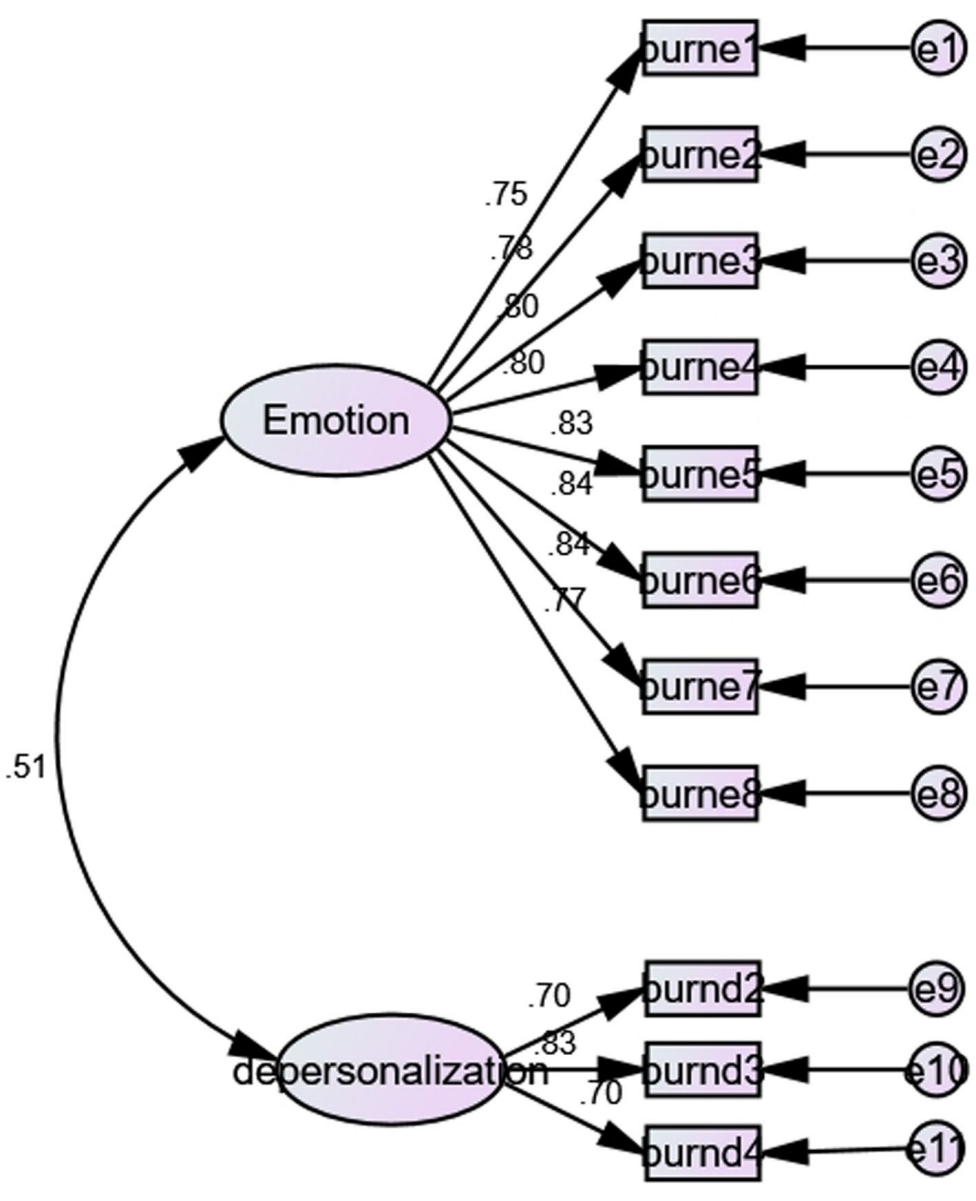
The results of CFA.

In this study, a confirmatory factor analysis (CFA) was conducted using AMOS 21.0 to examine the two-factor structural equation model of social burnout. The two dimensions, “emotional exhaustion” and “depersonalization,” were represented by 11 items (See Figure 1).

The maximum likelihood method was used to estimate the structural equation model, which required the data to be normally distributed. The normal distribution of the data was related to the skewness and kurtosis values of the test measured variables. It was generally believed that if the absolute value of skewness is less than 2 and the absolute value of kurtosis is less than 5 (Ghiselli et al., 1981), the data was normally distributed. The skewness and kurtosis distribution of the sample data in this paper were shown in Supplementary Appendix S1. It can be seen that the absolute value of skew was less than 2 and the absolute value of kurtosis was less than 5, and the data was normally distributed. Furtherly, the results of CFA showed that the two dimensions had an excellent fit: (χ^2^/df = 3.23, RMSEA = 0.08, NFI = 0.92, TLI = 0.92, and CFI = 0.94.)

### Phase 2: internal consistency

To determine the level of internal consistency of the social burnout scale, Cronbach’s alpha was computed using sample 4 and 5. Given that values of 0.70 or 0.75 are often used as cutoff value for Cronbach’s Alpha (Christmann and Aelst, 2002), we chose 0.75 was chosen as the lower-bound.

#### Participants

We used Group 1 as a sample in this step. This sample included 91 males and 332 females. There were 109 freshmen (25.80%), 210 sophomores (49.60%), 67 juniors (15.80%) and 37 seniors (8.80%). There were 367 (86.80%) students majoring in medicine and 56 (13.20%) students majoring in non-medicine.

#### Results

As is shown in Table 8, the results revealed that the “emotional exhaustion” dimension was 0.94, while that of the “depersonalization” dimension was 0.82, and the overall Cronbach’s α coefficient of the scale was 0.92. Both the reliability coefficient and the reliability coefficient of each dimension exceeded the stand of 0.70, which indicated that the social burnout scale developed in this study had good reliability (Ouyang et al., 2023).

**Table 8:** The results of reliability and validity test of social burnout scale.

Factor	Item number	Standardized load	Cronbach’s α	CR
Emotional Exhaustion	E7	0.84	0.94	0.93
	E6	0.83		
	E5	0.83		
	E4	0.83		
	E2	0.83		
	E3	0.82		
	E1	0.79		
	E8	0.73		
Depersonalization	D3	0.87	0.82	0.81

### Phase 3: convergent and discriminant validity

In terms of validity, this study mainly assessed the convergent validity and discriminant validity of the scale, using sample 4 and 5. Convergent validity refers to the items that measure the same variable falling on the same factor, emphasizing that the items that should have been under the same factor are indeed under the same factor. Discriminant validity represents the degree to which a latent variable differs from other latent variables. It means that a latent variable could account for more variance in the observed variables than other constructs within the conceptual. Technically, discriminant validity requires that “a test not correlate too highly with measures from which it is supposed to differ” (Farrell and Rudd, 2009). Convergent validity usually measured by the average variance extracted (AVE) and composite reliability (CR). These two indicators were calculated based on the standardized loadings of each item on its corresponding factor. While according to Fornell and Larcker (1981), discriminant validity could be assessed by AVE and shared variance. “For any two constructs, A and B, the AVE for A and the AVE for B both need to be larger than the shared variance (i.e., square of the correlation) between A and B″ (Farrell and Rudd, 2009).

#### Measures

*Social burnout* was measured with the 11-item scale developed in this study. Sample item was “Interpersonal interactions make me tired.” The internal consistency estimating for the scale was 0.922. Participants responded to the items on a 5-point scale ranging from *strongly disagree* (1) to *strongly agree* (5).

*Social fear* was measured with the 17-item scale developed by Connor et al. (2000). Sample item was “Fear of people in authority.” The internal consistency estimating for the scale was 0.845. Participants responded to the items on a 5-point scale ranging from *strongly disagree* (1) to *strongly agree* (5).

*Social anxiety* was measured with the 12-item scale developed by Sun et al. (2017). Sample item was “I am afraid that others will not like me.” The internal consistency estimating for the scale was 0.890. Participants responded to the items on a 5-point scale ranging from *strongly disagree* (1) to *strongly agree* (5).

#### Participants

We used Group 1 as a sample in this step again.

#### Results

Firstly, we calculated the correlation coefficient between social burnout and social fear, as well as social anxiety. We expected the positive correlation to be significant but not so high. As predicted, both social fear and social anxiety had moderate positive correlation with social burnout (r = 0.63, *p* < 0.01; r = 0.56, p < 0.01). The results provided initial evidence in support of the distinctness of the social burnout scales.

Secondly, as shown in Table 8, the Fornell and Larcker (1981) test showed that, the AVE for emotional exhaustion and depersonalization were 0.60 and 0.59, and the CR values were 0.93 and 0.81, respectively. Both AVE values were greater than the critical value of 0.50, and both CR values were higher than the critical value of 0.70, indicating good convergent validity. Besides, both the AVE for emotional exhaustion and depersonalization were larger than square of the correlation between them, indicating good discriminant validity of the scale.

### Phase 4: nomological network and criterion-related validity

We also verify the nomological network and criterion-related validity of the social burnout scale using several theoretical hypotheses (see Phase 4 for details).

#### Nomological network

Social skills which are defined as “different classes of social behavior within the individual repertoire to deal appropriately with demands of interpersonal situations” (Gomes and Pereira, 2014), are often considered to be critical to the processes of social adjustment and functioning of individuals. A variety of studies have proposed that social skills are negatively associated with negative feelings about social interaction (e.g., social anxiety; Hsu et al., 2012; Mokuolu, 2013). For example, social skills are negatively related to components of burnout in medical residents (Pereira-Lima and Loureiro, 2015). It has been argued that, social skills may act as proactive factors in preventing burnout and other mental health problems such as anxiety and depression (Pereira-Lima and Loureiro, 2015). Therefore, in this study, social skills were selected for criterion-related validity test, and the hypothesis was:

*H1*: Social skills have a significant positive effect on social burnout.

Interpersonal trust refers to a generalized expectation of the person with whom an individual establishes in the process of interpersonal interaction (Mayer et al., 1995). It is not only an important factor in college students’ interpersonal communication, but also a common recognition of the reliability of others’ words and deeds in the interaction between individuals and others. As a basis for building a good interpersonal relationship, interpersonal trust may be a major cause of emotional problems like social anxiety, social fear, and so on. For example, the relevant studies have found that interpersonal trust directly regulates the social anxiety of college students (Tian, 2005; He, 2022). A few researches suggested that some interpersonal problems were greatly affected by the interpersonal trust of individuals (Xu et al., 2017; Lai et al., 2019). Accordingly, we advanced the following hypothesis.

*H2*: Interpersonal trust have a significant positive effect on social burnout.

#### Measures

*Social burnout* was measured separately with the 11-item scale developed in this study. Sample item was “Interpersonal interactions make me tired.” The internal consistency estimating for the scale was 0.90. Participants responded to the items on a 5-point scale ranging from *strongly disagree* (1) to *strongly agree* (5).

*Social skills* were assessed with the 11-item scale developed by Shen et al. (2007), and the internal consistency estimated for the scale was 0.87. Sample item was “I am good at dealing with anyone I meet.” Respondents used a 5-point scale ranging from strongly disagree (1) to strongly agree (5) to respond to those items.

*Interpersonal trust* was assessed with the 10-item scale developed by Rotter (1967) and modified by Ding and Peng (2020). The internal consistency estimated for the scale was 0.70. Sample item was “In dealing with strangers one is better off to be cautions until they have provided evidence that they are trustworthy.” Respondents used a 5-point scale ranging from strongly disagree (1) to strongly agree (5) to respond to those items.

##### Control variables

Since previous studies had found that gender, grade, and major were factors affecting burnout, this study controlled for these three variables. For example, a meta-analysis found that women were likelier to report the emotional exhaustion burnout component than men, whereas men were likelier to report the depersonalization component of burnout than women (Purvanova and Muros, 2010). So, we controlled the variable of gender. It’s also found that burnout syndrome levels in college students varied between 8.00 and 56.90%, and this variation was associated with the specialty that a university student pursued (Rosales-Ricardo et al., 2021). Especially, distress during medical school could lead to burnout, which would force medical students’ well-being. Students rotating on hospital wards and those required to stay for overnight call were also more likely to experience burnout. Accordingly, we controlled for participants’ major as medicine and non-medicine. In addition, different grades could also affect burnout (IsHak et al., 2013). The scores on the emotional energy subscale showed a small rise from grade 1 to grades 4 and 5 (Guthrie et al., 1998). Accordingly, we controlled for participants’ grade.

#### Participants

We used sample 6 in this step. Sample 6 was an independent, multi-wave data sample. A total of 233 participants completed the first wave. One week later, 213 participants completed the second wave (91.40% response rate). This sample included 54 males and 159 females. There were 60 freshmen (28.20%), 106 sophomores (49.80%), 37 juniors (17.40%) and 10 seniors (4.70%). There were 191 (89.70%) students majoring in medicine and 22 (10.30%) students majoring in non-medicine.

#### Results

Correlations for sample 6 are shown in Table 9.

**Table 9:** Descriptive statistics and correlations for sample 6.

Variable	M	SD	1	2	3	4	5	6
(1) Gender	1.75	0.44	1					
(2) Grade	1.99	0.82	−0.06	1				
(3) Major	1.10	0.31	−0.05	0.34**	1			
(4) SB	2.53	0.64	−0.08	−0.02	−0.07	1		
(5) SS	3.00	0.51	−0.12	0.1	0.07	−0.39**	1	
(6) IT	2.70	0.47	0.07	0.01	0.12	−0.30**	0.21**	1

*n* = 213; SB, social burnout; SS, social skills; IT, interpersonal trust. *p < 0.05; **p < 0.01; ***p < 0.001.

As predicted in H1, social skills were negatively related to social burnout (r = −0.39, *p* < 0.01). Besides, as predicted in H2, interpersonal trust was negatively related to social burnout (r = −0.30, *p* < 0.01).

### Criterion-related validity

Life satisfaction is a subjective evaluation or judgment of an individual’s current quality of life (Kim et al., 2020), and it is also an important indicator to measure the level of individual mental health. Prior studies have demonstrated the negative relationship between emotional burnout and life satisfaction because of the health problems brought by it (Khamisa et al., 2015; Xu and Wang, 2023). According to the conservation of resources theory, burnout directly affects health outcomes by depleting resources needed for coping, resulting in a negative state characterized by exhaustion, fatigue, somatization, and social withdrawal. Accordingly, we advance the following hypothesis.

*H3*: Social burnout has a significant negative effect on life satisfaction.

#### Measures

*Social burnout* was measured separately with the 11-item scale developed in this study.

*Life satisfaction* was measured following the practice of Zhou et al. (2023) and two items of Van Damme’s life satisfaction scale were used. Sample item was “Overall, you are satisfied with your current school life.” Respondents used a 4-point scale ranging from strongly disagree (1) to strongly agree (4) to respond to those items.

#### Participants

We also used sample 6 in this step.

#### Results

To test H3, a regression analysis was conducted and the results were presented in Table 10. As predicted, social burnout had a significant negative effect on life satisfaction (*β* = −0.253, *p* < 0.001).

**Table 10:** The results of regression analysis.

Variable		Life satisfaction	
		Model 1	Model 2
Control variable	Gender	−0.10	−0.12
	Major	0.03	0.01
	Grade	−0.16	−0.16
Independent variable Social burnout			−0.25***
R^2^		0.03	0.10
△R^2^		0.03	0.06
F		2.27	14.56***

## Discussion

This study endeavored to define the concept of Social Burnout and develop a measurement tool for it.

As Bakker and Demerouti (2002) pointed out, burnout was not only limited to the service industry but could also occur in various professional fields. Recently, the phenomenon of burnout has been disclosed more and more among college students (Alkhamees et al., 2020; Rodrigues et al., 2020; Kaggwa et al., 2021; Rosales-Ricardo et al., 2021; Tlili et al., 2021). The current research on students’ burnout mainly refers to academic burnout, and one of the most important phenomena has been neglected, that is, social burnout. What’s more, the existing measurement of burnout in the college students mainly modified the maturity scale, which limited the development of related research. This study adopts the Corbin and Strauss (2008)’s research paradigm, including data collection, open coding, axial coding, selective coding and theoretical saturation testing, to conduct qualitative research on social burnout. In this study, a sample of 65 students were interviewed by semi-structured interviews to obtain first-hand data, and then an integrated database was obtained by combining the literature data. After that, the data is encoded according to Strauss (1987) proceduralised grounded theory, which includes open coding, axial coding and selective coding. Through data coding, it is found that social burnout does exist among college students, which mainly includes two types: emotional exhaustion and depersonalization.

Using four samples, we validated the social burnout scale in this study. We developed a 11-item social burnout scale and established the initial reliability and validity of this new scale. In line with prior researches (Densten, 2001; Galanakis et al., 2009), our results supported the two-factor structure of burnout. Although Maslach and Jackson (1981a,b) asserted that, emotional exhaustion, depersonalization, and low personal accomplishment are separated factors of the burnout construct, and a large number of studies support the three-factor structure of MBI (Worley et al., 2008). The MBI has also been criticized for its inconsistent factorial validity, because one- (Leiter, 1993), two - (Galanakis et al., 2009), four -(Firth et al., 1985), or five-factor (Densten, 2001) structures had also been found. This study verified it again that the three-factor structure was not always replicated. Indeed, some researches had confirmed that the core of MBI-measured burnout was more accurately captured by exhaustion and depersonalization/cynicism (Schaufeli et al., 2009; Beer and Bianchi, 2017; Prata et al., 2021).

The reliability of SBS was very good: the reliability of the two subscales ranged from 0.82 (depersonalization) to 0.94 (emotional exhaustion). Similar results were found in other studies (Obregon et al., 2020; Schaufeli et al., 2020), wherein Cronbach’s alpha for depersonalization and emotional exhaustion were > 0.80, implying good internal consistency. However, compared to Obregon et al. (2020), whose Cronbach’s alpha coefficients for emotional exhaustion was 0.834, our results showed a more preferable internal consistency. Furthermore, the CFA model’s indexes demonstrated that the SBS’s two-dimensional structure achieve an adequate fit for the data.

In terms of convergent validity and discriminant validity, both social fear and social anxiety had moderate positive correlation with social burnout (r = 0.63, *p* < 0.01; r = 0.56, *p* < 0.01), which provided the initial evidence in support of the distinctness of the social burnout scales. In fact, as pointed by (Hu et al., 2020), anxiety, burnout, and fear were different variables, and our study again validates this assertion. Furthermore, the Fornell and Larcker (1981) test showed that both AVE values of emotional exhaustion (0.60) and depersonalization (0.59) were greater than the critical value of 0.50, and both CR values (emotional exhaustion: 0.93; depersonalization: 0.81) were higher than the critical value of 0.70, indicating good convergent validity. Besides, both the AVE for emotional exhaustion and depersonalization were larger than square of the correlation between them, indicating good discriminant validity of the scale. Just as Schaufeli et al. (2020) asserted, the results further suggested that the construct of social burnout, as measured by SBS, could be discriminated from other well-being constructs.

The nomological network and criterion-related validity were also verified. The negatively correlations between social skills as well as interpersonal trust and social burnout was statically significant, suggesting that the SBS was related to important psychological variables. The demand-resource model of burnout has suggested that burnout occurs when an individual’s resources cannot meet the demand (Demerouti et al., 2001). But when the individual’s resources were supplemented, they could better cope with burnout. Social skills and interpersonal trust were both important personal resources that can provide support to an individual. According to Kiema-Junes et al. (2020), social skills could increase one’s competence and emotional and behavioral adjustment, which might be associated with lower risk of burnout. Besides, interpersonal trust had also been verified to be a supporting variable for burnout. The lower the interpersonal trust, the higher the severity of stress. Our study supported the prior view again that there was a negative correlation between interpersonal trust and burnout (Bobbio, 2012; Chudzicka-Czupała et al., 2022).

What’s more, the significant negative effect of social burnout on life satisfaction showed the criterion-related validity of SBS. These results were consistent with the asertion that burnout was a striking social and health problem occurred in situations where people developed intense relationships. This syndrome linked with several forms of adverse influence on the physical and mental health, as well as quality of life (Meneguin et al., 2023).

Our findings have practical implications. Considering that student burnout is a serious health issue that has been widely investigated in recent decades (Fiorilli et al., 2022), educational institutions and faculty members must prioritize identifying various types of burnout in students and providing more mental health support and assistance to students. Effective and easily accessible screening tools can support students’ mental health care. SBS developed in this study is an effective screening tool for emotional exhaustion and depersonalization in interpersonal communication.

## Limitations and future research directions

There are some limitations with our studies that need to be considered.

First, a potential limitation was the use of a single-wave sample in the construct validation (i.e., sample 4 and 5), which could lead to common method bias in this sample. Whatever possible, we did use a multi-wave data in sample 6, trying our best to minimize method bias. Our goal was to build confidence that the social burnout scale was appropriate for the real world.

Second, while the study followed standard procedures for developing a measurement scale of social burnout and tested the criterion-related and predictive validity using social skills, interpersonal trust and life satisfaction, it should be noted that there may be varied factors could be chosen. For example, a large number of studies have confirmed the negative correlation between burnout and job performance (Cordes and Dougherty, 1993; Leiter et al., 1994; Lu et al., 2006; Liu and Yang, 2019). Some studies have also found that stressful events and personal traits have a significant impact on burnout (Liu and Yang, 2019; Chen et al., 2023). To enhance the construct validity of the scale, future studies should examine the relationship between these variables and social burnout.

Finally, we have not conducted a cross-cultural measurement invariance test. The conclusion obtained in the Chinese context may not be suitable for other contexts. Future studies may replicate the process of this study in other cultural contexts.

## Conclusion

Although the assertion that burnout can occur in all areas that give people a sense of value and meaning has been allowed to persist for decades, and the study of college students’ burnout is not a new topic, researchers have tended to use existing scales rather than develop more applicable ones. The evidence that we have presented is consistent with our argument that the classical burnout scale may not suitable for measuring social burnout, suggesting that social burnout should be studied on its own, independent of general burnout. Overall, this study deepens our understanding of the burnout family and finds new members of it. We hope that the social burnout scale developed in this paper will encourage and facilitate the systematic study of this symptom.

## Data availability statement

The raw data supporting the conclusions of this article will be made available by the authors, without undue reservation.

## Ethics statement

Ethical review and approval was not required for the study on human participants in accordance with the local legislation and institutional requirements. All procedures were in accordance with the ethical standards of the national research committee. Written informed consent from the participants was not required to participate in this study in accordance with the national legislation and the institutional requirements. Prior to conducting the study, we provided respondents with a cover letter indicating their willingness to participate and keeping their answers confidential. Respondents participated voluntarily and were free to withdraw at any point in time.

## Author contributions

JW: Conceptualization, Data curation, Formal analysis, Writing – original draft. CL: Conceptualization, Writing – original draft, Data curation, Formal analysis. LC: Investigation, Writing – review & editing. QL: Conceptualization, Data curation, Formal analysis, Writing – original draft, Funding acquisition, Writing – review & editing. GL: Investigation, Methodology, Writing – review & editing.
