# Supplementary material for: Development of the social burnout scale for college students
Source: Front Psychol. 2024 Mar 21;15:1295755. doi: 10.3389/fpsyg.2024.1295755 (PMC10991744; doi:10.3389/fpsyg.2024.1295755)
Supplement: Supplementary file 1 [file Table_1.DOCX]

**Appendix 1 Skewness and kurtosis analysis of sample 5**

|  | **Skewness** | | **Kurtosis** | |
| --- | --- | --- | --- | --- |
|  | Statistic | Std. Error of Skewness | Statistic | Std. Error of Kurtosis |
| E1 | 0.19 | 0.17 | -0.61 | 0.33 |
| E2 | 0.44 | 0.17 | -.035 | 0.33 |
| E3 | 0.50 | 0.17 | 0.32 | 0.33 |
| E4 | 0.30 | 0.17 | -0.13 | 0.33 |
| E5 | 0.51 | 0.17 | 0.07 | 0.33 |
| E6 | 0.28 | 0.17 | -0.54 | 0.33 |
| E7 | 0.29 | 0.17 | -0.64 | 0.33 |
| E8 | 0.43 | 0.17 | -0.18 | 0.33 |
| D2 | 0.68 | 0.17 | 1.05 | 0.33 |
| D3 | 0.65 | 0.17 | 1.48 | 0.33 |
| D4 | 0.80 | 0.17 | 1.22 | 0.33 |
